# Assessment of Continuity of Care among Patients with Multiple Chronic Conditions in Italy

**DOI:** 10.1371/journal.pone.0154940

**Published:** 2016-05-03

**Authors:** Francesco Napolitano, Paola Napolitano, Luca Garofalo, Marianna Recupito, Italo F. Angelillo

**Affiliations:** Department of Experimental Medicine, Second University of Naples, Via Luciano Armanni, Naples, Italy; University of Naples Federico II, ITALY

## Abstract

The aims of the present study were to evaluate the extent of continuity of care and to investigate its association with several factors among a sample of outpatients with chronic diseases in Italy. The survey was conducted, using face to face interview, from March to December 2014 in a random sample of 633 outpatients with chronic conditions who were going in cardiology, metabolic disorders, and respiratory ambulatory center of four hospitals. A multivariate ordered logistic regression model was used to identify factors associated with the outpatients continuity of care. The mean of the Bice-Boxerman continuity of care (COC) index related to the entire sample was 0.44, and 27.9%, 58.4%, 13.7% had a low, intermediate, and high value of the index based on the tertiles of the distribution. The results of the ordered logistic regression analysis showed that female patients, those older, those who had a lower score of Katz Index of independence in activities of daily living, those who had a lower Charlson et al. comorbidity score, and those who had no hospitalization in the last year, were significantly more likely to have a higher value of the COC index. Patients who had completed a secondary school education had significantly lower odds of having a high value of COC index in comparison to patients with a college degree educational level. Policy makers and clinicians involved in the care of patients should implement comprehensively and efficiently efforts in order to improve the continuity of care in patients with chronic diseases.

## Introduction

It is widely recognized that continuity of care is an essential element for the delivering of high-quality of health care and it is of particular importance for patients with chronic conditions or for those who suffered from multiple comorbidities. Indeed, many of these patients require chronic and complex management from a variety of health care professionals in multiple settings and, therefore, they are one of the major users of health care services [1,2]. Moreover, it is well known that a poor social support and comorbidities are negatively associated with functional status and mortality particularly in elderly patients [3–5]. The increasing prevalence of patients with one or more chronic conditions and the complexity of the treatments, with a higher number of medications per day, of medical specialist’s visits, and of accesses at hospital and emergency facilities, have accentuated the patients’ expectations and consequently they tended to see several physicians in different settings in order to satisfy their health needs. Continuity of care could result in a reduction of health care costs with reducing fragmentation of care of the patients, better outcomes for their diseases, and a higher level of their satisfaction [6–13].

Although several studies have been conducted to measure the continuity of care and to identify its predictors for patients with a single chronic condition such as cardiovascular diseases [12,14,15], diabetes [14–18], mental illness [19], and chronic obstructive pulmonary disease [12,14,20], there have been few attempts until recently to study patients with multiple chronic conditions [21–23].

Furthermore, as per our knowledge, previously published studies evaluating the continuity of care in outpatient settings among those who had chronic diseases in Italy have been all but absent. Therefore, this current study was designed to determine the extent of continuity of care and to investigate its association with several factors among a sample of outpatients with chronic diseases in Italy.

## Materials and Methods

This observational cross-sectional study was conducted in the cities of Caserta and Naples, Italy, from March until December 2014. The study design and the methodology are described in detail elsewhere [24]. Briefly, the sample was selected with a two stage sampling method. Firstly, from the list of public hospitals in the geographic area, four hospitals have been selected using a single random sampling method. In the second stage, 770 patients have been selected using a single random sampling method among outpatients with chronic conditions attending the ambulatory centers of cardiology, metabolic disorders, and respiratory of the selected hospital. Patients were eligible for the survey if they were 18 or above years of age and if they had at least one chronic disease. The patients with mental illness, cognitive impairment, or those who were unable to be interviewed have been excluded. Participants were informed that all information gathered would be anonymous and handled confidentially; no identifying information was collected. Participation was completely voluntary; written informed consent was obtained from individual respondents after a complete description of the study before the questionnaires were administered to them. Patients were free to participate or not in the survey.

The sample size was calculated based on an estimated rate of the population with high continuity of care of 35%, chosen as the primary endpoint, a confidence level of 95%, an accepted precision of 5%, and a design effect of two. The required sample size was estimated to be approximately of 700 patients. To allow for 90% participation rate, 770 patients were the final sample size.

The respondents underwent a face-to-face interview with a questionnaire during routine outpatient consultations. Names were not required on the questionnaires to maintain confidentiality. Respondents were interviewed before their clinic consultations using a questionnaire divided into five parts including questions sealing with: (a) socio-demographic and clinical profile characteristics (age, gender, level of education, marital status, employment status, number and type of chronic conditions (heart diseases, metabolic disorders, respiratory diseases), pre-admission performance-based measure of independence in activities of daily living using the Katz Index [25], comorbidities measured with the Charlson *et al*. Comorbidity Index [26]; (b) health-care services utilization in the previous twelve months (number of specialists’ and of general practitioners’ visits, hospital admissions, emergency department visits); (c) type and number of medications used; (d) medications adherence; and (e) main source of information on the use of medicines and the needs of additional information about their chronic conditions.

Adherence to medications was assessed using the Morisky Medication Adherence 4-items Scale (MMAS-4) [27] over the 4 weeks preceding their medical visit. Continuity of care was assessed using the Bice-Boxerman continuity of care (COC) index [28] which has measured for each patient the number of different physicians seen and the number of visits for an episode of illness to each physician in each year during the study period. The COC emphasizes the distribution of visits to each health-care physician that the patient visited. This index measures the degree to which patient visits are dispersed among different physicians. The value of the COC index ranges between 0 and 1, with higher values representing a lower dispersion and a higher continuity of care. If a patient has all ambulatory care visits with the same physician, the index is equals to 1, representing a lower dispersion and a higher continuity of care, whereas if visits are with all different physicians, the index becomes 0, representing a higher dispersion and a lower continuity of care. When calculating the COC index, surgical visits and outpatient surgery have been excluded.

The questionnaire was tested in a pilot study among a sample of 25 outpatients before the present study, to evaluate the comprehensibility and the validity.

The study protocol and the questionnaire were approved prior to conducting the interviews by the Ethical Committee of the Second University of Naples.

### Statistical analysis

The statistical analysis was performed in two steps. First, chi-square test, Fisher’s exact test, and Student’s *t*-test test were used to assess the statistical significant associations between the continuity of care as outcome of interest and the different explanatory categorical and continuous variables, respectively. Variables with a *p*-value less than or equal to 0.25 from the univariate analysis, were considered eligible for inclusion into the multivariate regression analysis. In a second step, a multivariate ordered logistic regression analysis was developed using outpatients continuity of care as the outcome variable to identify the independent predictors of high continuity of care. The ordered logistic regression analysis has been performed, although the outcome was originally a continuous variable, since the interest was to measure the difference between groups of patients according to the value of the COC index. Therefore, the COC index was transformed into a categorical variable and divided into three groups based on the tertiles of the distribution of the scores as low with a value between 0–0.33, intermediate between 0.34–0.66, and high between 0.67–1. A stepwise method has been used to include in the final regression model only the variables that provide a significant explanation of the outcome. The choice for the inclusion and elimination of the variables in the model were respectively *p*-values of 0.2 and 0.4. The contribution of each exploratory variable in the multivariate ordered logistic regression analysis was expressed as odds ratio (OR) and a 95% confidence interval (CI). The multivariate ordered logistic regression model built to identify factors associated with the outpatients continuity of care was adjusted for the following independent characteristics of each respondent: gender (male = 0; female = 1), age (continuous, in years), marital status (single/separated/divorced/widowed = 0; married = 1), number of cohabiting (continuous), educational level (three categories: primary school or lower = 1; secondary school = 2; college degree or higher = 3), occupation (unemployed = 0; employed = 1), score of the Katz index of pre-admission performance-based measure of independence in activities of daily living (continuous), score of the Charlson *et al*. comorbidity index (continuous), attending the emergency department in the last year (no = 0; yes = 1), hospital admission in the last year (no = 0; yes = 1), number of pills per day (continuous), medication adherence (no = 0; yes = 1), physicians as source of information (no = 0; yes = 1), and need of additional information about their chronic diseases (no = 0; yes = 1).

In all analyses a level of significance equal or below 5% based on two-sided was considered to be statistically significant. Statistical analysis was carried out with the statistical software package Stata version 10.1 [29].

## Results

A total of 770 eligible patients were recruited, with 633 completing their interview amounting to a response rate of 82.2%. The principal characteristics of the overall sample, the frequency distribution according to the different values of the COC index and the results of the bivariate analyses are summarized descriptively in Table 1. The sample was balanced in terms of gender, the average age was 63.2 years, the median Katz index of pre-admission performance-based measure of independence in activities of daily living was 6, the Charlson *et al*. comorbidity score was two, the median of number of chronic conditions was 3, the median number of drugs and of pills taken per day were respectively 3 and 4. The patients were mainly affected by hypertension (81.8%), diabetes (34.4%), chronic obstructive pulmonary disease (17.1%), and coronary artery disease (15.3%). Regarding the use of healthcare services in the last year, all the sample had at least one visit to the specialist with a median visits of 3, almost all had at least one visit by their general practitioner, and the rate of emergency department visit and hospital admissions were respectively 31.4% and 20.7%. Among those who had a hospital admission in the last year, 15.4% had more than one hospitalization. Approximately 40% of respondents reported themselves as being adherent to medications over the 4 weeks preceding their medical visit according to the Morisky score of 0.

**Table 1:** Characteristics of the overall sample according to the different COC groups.

	Total*n* = 633		Low COC group*n* = 177		Intermediate COC group*n* = 370		High COC group*n* = 86		*p*
	*n*	%	*n*	%	*n*	%	*n*	%	
*Gender*									0.021
Male	327	51.7	107	60.4	180	48.6	40	46.5	
Female	306	48.3	70	39.6	190	51.4	46	53.5	
*Age (years)*	63.2±11.9(18–96)*		62.7±12.1(24–96)*		63.5±11.9(18–88)*		62.9±11.9(32–88)*		0.715
*Educational level*									0.133
Primary school or lower	229	36.2	53	29.9	142	38.4	34	39.5	
Secondary school	198	31.3	60	33.9	118	31.9	20	23.3	
College degree or higher	206	32.5	64	36.2	110	29.8	32	37.2	
*Marital status*									0.842
Married	510	80.5	140	79.1	300	81.1	70	81.4	
Other	123	19.5	37	20.9	70	18.9	16	18.6	
*Katz Index of Independence in Activities of Daily Living^a^*	5.4±1.3(0–6)*		5.5±1.2(0–6)*		5.4±1.3(0–6)*		5.5±1.2(1–6)*		0.981
*Charlson et al*. *comorbidity score^b^*	2.5±1.3(1–8)*		2.7±1.4(1–7)*		2.8±1.4(1–7)*		1.9±0.9(1–5)*		*<*0.001
*Number of chronic conditions*	2.8±1.3(1–7)*		3.1±1.2(1–6)*		2.8±1.4(1–7)*		2.2±1.1(1–5)*		*<*0.001
*General practitioners’ visits in the last year*	6.4±4.1(0–20)*		3.9±2.5(0–15)*		7.2±4(0–20)*		8.1±4.9(0–20)*		<0.001
*Medical specialist’s visits in the last year*	3.7±2.2(1–14)*		5±2.3(1–14)*		3.6±2(1–10)*		1.6±1.2(1–6)*		*<*0.001
*Pills per day*	5.5±3.7(1–18)*		6.1±3.3(1–16)*		5.6±3.9(1–18)*		4.1±3.5(1–13)*		*<*0.001
*Hospitalizations in the last year*									0.001
No	502	79.3	128	72.3	294	79.5	80	93.1	
Yes	131	20.7	49	27.7	76	20.5	6	6.9	
*Emergency department visits*									0.111
No	434	68.6	112	63.3	257	69.5	65	75.6	
Yes	199	31.4	65	36.7	113	30.5	21	24.4	

* Mean ± standard deviation (range)

a Range from 0 to 6 with highest scores indicate complete independence

b Highest scores indicate greater comorbidity

The mean of COC index related to the entire sample was 0.44, and respectively the 27.9%, the 58.4%, and 13.7% of the participants had a low, intermediate, and high value of the index based on the tertiles of the distribution. The bivariate analysis revealed that seven variables were significantly associated with the level of outpatients’ continuity of care: gender, Charlson *et al*. comorbidity score, number of chronic conditions, number of general practitioner visits in the last year, number of specialist visits in the last year, number of pills per day, and hospital services use.

The results of the multivariable ordered logistic regression analysis to model the likelihood of outpatients’ continuity of care are presented in Table 2.

**Table 2:** Ordered logistic regression analyses performed to identify the association of independent variables with the outpatients continuity of care.

Variable	OR	SE	95% CI	*p* value
Model. Outpatients continuity of care (sample size n = 633)				
Log likelihood = -564.29, χ^2^ = 63.2 (15 df), *p*<0.0001				
Charlson et al. comorbidity score	0.71	0.06	0.59–0.84	<0.001
Educational level				
Primary school or lower	1*	-	-	-
Secondary school	0.57	0.12	0.38–0.86	0.008
College degree or higher	0.65	0.15	0.41–1.03	0.069
Hospitalizations in the last year	0.56	0.1	0.37–0.86	0.008
Age	1.02	0.01	1.01–1.04	0.036
Gender	1.43	0.25	1.02–2.01	0.038
Katz Index of independence in activities of daily living	0.85	0.06	0.74–0.99	0.038
Occupation	1.44	0.29	0.96–2.15	0.076
Marital status	1.47	0.32	0.95–2.26	0.081
Physicians as source of information	0.79	0.13	0.57–1.09	0.157
Visits in emergency department in the last year	0.84	0.15	0.59–1.19	0.366
Number of cohabiting	0.95	0.07	0.82–1.11	0.509
Number of pills per day	0.98	0.03	0.92–1.05	0.621
Need of additional information about their chronic diseases	1.07	0.18	0.76–1.52	0.666
Medication adherence	0.97	0.17	0.68–1.39	0.871

* Reference category

The results of the model partially confirmed those obtained in the bivariate analysis. Of the several demographic characteristics of the patient, gender, age, and educational level resulted statistically significant associated with the COC index at a significance level of 5%. The odds of being in the high COC group versus the combined low and intermediate groups are about 1.43 times greater for female compared to male (OR = 1.43, 95% CI = 1.02–2.01). Each additional year of age is associated with 2% increase in the odds of reporting high COC value versus the combined low and intermediate groups (OR = 1.02, 95% CI = 1.01–1.04). Patients who had completed a secondary school education had significantly lower odds of having a high values of COC index in comparison to patients with a college degree educational level (OR = 0.57, 95% CI = 0.38–0.86). Patients who have had no hospitalization in the last year (OR = 0.56, 95% CI = 0.37–0.86), those who had a lower score of Katz Index of independence in activities of daily living (OR = 0.85, 95% CI = 0.74–0.99), and a lower Charlson et al. comorbidity score (OR = 0.71, 95% CI = 0.59–0.84) were significantly more likely to have a higher value of the COC index.

Regarding the information about the chronic diseases of patients, the majority of sample (53.1%) receives information by physicians about the need to undergo regular clinics checks, and only 30.5% said that they need additional information about the medications for their condition.

## Discussion

The study described here, the first face-to-face questionnaire survey to be performed in Italy, is evaluating the continuity of care in outpatient settings among patients who had chronic diseases and the predisposing and enabling factors to evaluate the determinants of continuity of care.

A number of difficulties arise in a direct and sound comparison with other similar previous published reports from various countries and this study cannot rule out the possibility that the observed differences are due to different setting or population studied, time periods, methods of assessment, ways of reporting, and access of the patients to health care or the healthcare system. This study population had a higher continuity of care compared with other studies, since the mean COC index (0.44) was higher that the values of 0.31 reported in an observational retrospective cohort group of patients older than 65 years in the United States [30] and of 0.37–0.39 in a follow-up study in Taiwan in patients who were first diagnosed with type 2 diabetes [16]. Moreover, this research is in line with those of some previous other studies worldwide with a value of 0.49 that has been observed in a follow-up study in Taiwan in patients at the site level [22] and in patients receiving long-term frequent care in Norway [31], 0.5 and 0.55 in the United States in sample of Medicare beneficiaries experiencing a 12-month episode of care respectively for type 2 diabetes mellitus and for congestive heart failure [12]. By contrast, the value of the Continuity of Care Index here is lower than those observed in the United States in a retrospective cohort study among seniors with 3 or more chronic conditions with a mean value of 0.6 [23], in Korea among elderly people where the mean value was 0.735 for hypertension, 0.709 for diabetes mellitus, 0.7 for chronic obstructive pulmonary disease, 0.663 for asthma [32], and 0.752 for an adult study population with type 2 diabetes [33], and 0.74–0.76 with a primary care physician in the French general population [34].

One of the main objectives of the present investigation was to identify the factors influencing continuity of care. Interestingly, multivariate regression analysis, used to identify the characteristics associated with different levels of continuity, found that a number of relevant factors were more likely to significantly predict the continuity of care. Of the patients’ socio-demographic characteristics, gender, age, and educational level were found to be significantly associated with the outcome with women, older patients, and those who had completed a secondary school education were likely to be in the high COC group. Similarly, in a study conducted in the United States patient characteristics associated with lower odds of continuity were male sex and having a low socioeconomic status [35]. Another interesting finding is that not being admitted into hospital ward in the last year was positively associated with a higher value of the COC index. A similar finding has been observed in the already mentioned studies conducted in Korea and in the United States who found that higher continuity of care scores were significantly associated with a reduced risk of hospitalization [32] and inpatient admission [12,23]. Moreover, in Canada a group of family practice patients 65 years of age or older with diabetes with higher continuity of care index scores were significantly less likely to experience hospitalization [36] and in the United States a large sample of enrolled fee-for-service Medicare beneficiaries older than 65 years a higher continuity was associated with a lower rate of preventable hospitalization [37]. The findings of the present study should be interpreted within the context of certain potential limitations. First, the cross-sectional design of this study prevents us, as with surveys of this nature, from making any robust ascertainment of causal relationships. Second, the results obtained in this questionnaire survey were gathered by face-to-face interview and based only on the participant’s report. An inherent limitation of all face-to-face interview and self-reported questionnaires is that the accuracy of the results was heavily dependent on the honesty and recall ability of the respondents and might generate social desirable answer. It has attempted to circumvent this issue by ensuring patients with complete confidentiality and anonymity in the data collection from questionnaire survey, which likely reduced the tendency of respondents to provide socially desirable answers with an underreporting or overreporting of the actual visits. The reported data from the survey were not confirmed by clinicians, patient records, or administrative claims data and, therefore, it is not known how accurately patients recall actual experience and patient reports of their behavior may not always correspond to their actual behavior. There may be recall error regarding previous visits, but any such effect is likely to be equally distribute in the sample. Despite these limitations, this research provides new and important information that contribute to the understanding of continuity of care and the main strengths are that this is first to be performed in this geographic area and the relatively high response rate.

In summary, the findings of this study suggest that policy makers and clinicians involved in the care of patients should implement comprehensively and efficiently efforts in order to improve the continuity of care in patients with chronic diseases.

## Supporting Information

S1 Questionnaire Questionnaire. (DOCX)

## References

[1] Steiner CA, Friedman B. Hospital utilization, costs, and mortality for adults with multiple chronic conditions, Nationwide Inpatient Sample, 2009. Prev Chronic Dis. 2013;10: E62 10.5888/pcd10.120292 23618542 PMC3652722

[2] Condelius A, Edberg AK, Jakobsson U, Hallberg IR. Hospital admissions among people 65+ related to multimorbidity, municipal and outpatient care. Arch Gerontol Geriatr. 2008;46: 41–55. 17403548 10.1016/j.archger.2007.02.005

[3] Nyqvist F, Nygård M, Steenbeek W. Social capital and self-rated health amongst older people in Western Finland and Northern Sweden: a multi-level analysis. Int J Behav Med. 2014;21: 337–347. 10.1007/s12529-013-9307-0 23584727

[4] Santini ZI, Koyanagi A, Tyrovolas S, Haro JM, Fiori KL, Uwakwa R, et al Social network typologies and mortality risk among older people in China, India, and Latin America: A 10/66 Dementia Research Group population-based cohort study. Soc Sci Med. 2015;147: 134–143. 10.1016/j.socscimed.2015.10.061 26575604

[5] Mazzella F, Cacciatore F, Galizia G, Della-Morte D, Rossetti M, Abbruzzese R, et al Social support and long-term mortality in the elderly: role of comorbidity. Arch Gerontol Geriatr. 2010;51: 323–328. 10.1016/j.archger.2010.01.011 20153534

[6] Ionescu-Ittu R, McCusker J, Ciampi A, Vadeboncoeur AM, Roberge D, Larouche D, et al Continuity of primary care and emergency department utilization among elderly people. Can Med Assoc J. 2007;177: 1362–1368. 18025427 10.1503/cmaj.061615 PMC2072991

[7] Cheng SH, Chen CC, Hou YF. A longitudinal examination of continuity of care and avoidable hospitalization: evidence from a universal coverage health system. Arch Intern Med. 2010;170: 1671–1677. 10.1001/archinternmed.2010.340 20937927

[8] Chen CC, Chen SH. Better continuity of care reduces costs for diabetic patients. Am J Manag Care. 2011;17: 420–427. 21756012

[9] Cheng SH, Hou YF, Chen CC. Does continuity of care matter in a health care system that lacks referral arrangements? Health Policy Plan. 2011;26: 157–162. 10.1093/heapol/czq035 20699348

[10] Russell D, Rosati RJ, Rosenfeld P, Marren JM. Continuity in home health care: is consistency in nursing personnel associated with better patient outcomes? J Healthc Qual. 2011;33: 33–39. 10.1111/j.1945-1474.2011.00131.x 22103703

[11] Russell D, Rosati RJ, Andreopoulos E. Continuity in the provider of home-based physical therapy services and its implications for outcomes of patients. Phys Ther. 2012;92: 227–235. Erratum in Phys Ther. 2012;92: 471. 10.2522/ptj.20110171 22074941

[12] Hussey PS, Schneider EC, Rudin RS, Fox DS, Lai J, Pollack CE. Continuity and the costs of care for chronic disease. JAMA Intern Med. 2014;174: 742–748. 10.1001/jamainternmed.2014.245 24638880 PMC4075052

[13] Shin DW, Cho J, Yang HK, Park JH, Lee H, Kim H, et al Impact of continuity of care on mortality and health care costs: a nationwide cohort study in Korea. Ann Fam Med. 2014;12: 534–541. 10.1370/afm.1685 25384815 PMC4226774

[14] Pollack CE, Hussey PS, Rudin RS, Fox DS, Lai J, Schneider EC. Measuring Care Continuity: A Comparison of Claims-based Methods. Med Care. Published online 2013 Dec. 10.1097/MLR.0000000000000018 PMC4101051 24309664

[15] Hanafi NS, Abdullah A, Lee PY, Liew SM, Chia YC, Khoo EM. Personal Continuity of Care in a University-Based Primary Care Practice: Impact on Blood Pressure Control. PLoS One. 2015;10: e0134030 10.1371/journal.pone.0134030 26214304 PMC4516235

[16] Chen CC, Tseng CH, Cheng SH. Continuity of care, medication adherence, and health care outcomes among patients with newly diagnosed type 2 diabetes: a longitudinal analysis. Med Care. 2013;51: 231–237. 10.1097/MLR.0b013e31827da5b9 23269110

[17] Cho KH, Lee SG, Jun B, Jung BY, Kim JH, Park EC. Effects of continuity of care on hospital admission in patients with type 2 diabetes: analysis of nationwide insurance data. BMC Health Serv Res. 2015;15: 107 10.1186/s12913-015-0745-z 25879858 PMC4393878

[18] Tsai HY, Chou YJ, Pu C. Continuity of care trajectories and emergency room use among patients with diabetes. Int J Public Health. 2015;60: 505–513. 10.1007/s00038-015-0671-1 25779687

[19] Fortney J, Sullivan G, Williams K, Jackson C, Morton SC, Koegel P. Measuring continuity of care for clients of public mental health systems. Health Serv Res. 2003;38: 1157–1175. 12968822 10.1111/1475-6773.00168 PMC1360938

[20] Lin IP, Wu SC, Huang ST. Continuity of care and avoidable hospitalizations for chronic obstructive pulmonary disease (COPD). J Am Board Fam Med. 2015;28: 222–230. 10.3122/jabfm.2015.02.140141 25748763

[21] Chan CL, You HJ, Huang HT, Ting HW. Using an integrated COC index and multilevel measurements to verify the care outcome of patients with multiple chronic conditions. BMC Health Serv Res. 2012;12: 405 10.1186/1472-6963-12-405 23157982 PMC3529188

[22] Cheng SH, Chen CC. Effects of continuity of care on medication duplication among the elderly. Med Care. 2014;52: 149–156. 10.1097/MLR.0000000000000042 24309666

[23] Bayliss EA, Ellis JL, Shoup JA, Zeng C, McQuillan DB, Steiner JF. Effect of continuity of care on hospital utilization for seniors with multiple medical conditions in an integrated health care system. Ann Fam Med. 2015;13: 123–129. 10.1370/afm.1739 25755033 PMC4369605

[24] Napolitano F, Napolitano P, Angelillo IF, Collaborative Working Group. Medication adherence among patients with chronic conditions in Italy. Eur J Public Health. 2016;26: 48–52. 10.1093/eurpub/ckv147 26268628

[25] Katz S. Assessing self-maintenance: activities of daily living, mobility, and instrumental activities of daily living. J Am Geriatr Soc. 1983;31: 721–727. 6418786 10.1111/j.1532-5415.1983.tb03391.x

[26] Charlson ME, Pompei P, Ales KL, McKenzie CR. A new method of classifying prognostic comorbidity in longitudinal studies: development and validation. J Chronic Dis. 1987;40: 373–383. 3558716 10.1016/0021-9681(87)90171-8

[27] Morisky DE, Green LW, Levine DM. Concurrent and predictive validity of a self-reported measure of medication adherence. Med Care. 1986;1: 67–74. 10.1097/00005650-198601000-00007 3945130

[28] Bice TW, Boxerman SB. A quantitative measure of continuity of care. Med Care. 1977;15: 347–349. 859364 10.1097/00005650-197704000-00010

[29] Stata Corporation. Stata Reference Manual Release 10.1: College Station, TX, USA 2007.

[30] Romano MJ, Segal JB, Pollack CE. The Association Between Continuity of Care and the Overuse of Medical Procedures. JAMA Intern Med. 2015;175: 1148–1154. 10.1001/jamainternmed.2015.1340 25984883 PMC5577558

[31] Gjevjon ER, Eika KH, Romøren TI, Landmark BF. Measuring interpersonal continuity in high-frequency home healthcare services. J Adv Nurs. 2014;70: 553–563. 10.1111/jan.12214 23869982

[32] Hong JS, Kang HC, Kim J. Continuity of care for elderly patients with diabetes mellitus, hypertension, asthma, and chronic obstructive pulmonary disease in Korea. J Korean Med Sci. 2010;25: 1259–1271. 10.3346/jkms.2010.25.9.1259 20808667 PMC2923791

[33] Hong JS, Kang HC. Continuity of ambulatory care and health outcomes in adult patients with type 2 diabetes in Korea. Health Policy. 2013;109: 158–165. 10.1016/j.healthpol.2012.09.009 23093021

[34] Leleu H, Minvielle E. Relationship between longitudinal continuity of primary care and likelihood of death: analysis of national insurance data. PLoS One. 2013;8: e71669 10.1371/journal.pone.0071669 23990970 PMC3750048

[35] Sharma G, Fletcher KE, Zhang D, Kuo YF, Freeman JL, Goodwin JS. Continuity of outpatient and inpatient care by primary care physicians for hospitalized older adults. JAMA. 2009;301: 1671–1680. 10.1001/jama.2009.517 19383958 PMC2771916

[36] Worrall G, Knight J. Continuity of care is good for elderly people with diabetes: retrospective cohort study of mortality and hospitalization. Can Fam Physician. 2011;57: e16–e20. 21252120 PMC3024182

[37] Nyweide DJ, Anthony DL, Bynum JP, Strawderman RL, Weeks WB, Casalino LP, et al Continuity of care and the risk of preventable hospitalization in older adults. JAMA Intern Med. 2013;173: 1879–1885. 10.1001/jamainternmed.2013.10059 24043127 PMC3877937

