# Supplementary material for: Assessment of Continuity of Care among Patients with Multiple Chronic Conditions in Italy
Source: PLoS One. 2016 May 3;11(5):e0154940. doi: 10.1371/journal.pone.0154940 (PMC4854373; doi:10.1371/journal.pone.0154940)
Supplement: S1 Questionnaire — (DOCX) [file pone.0154940.s001.docx]

**QUESTIONNAIRE**

**Respondent n° _________**  **Date ___________** **Ambulatory center ____________________**

**Section A.**

This section is designed to gather information about your socio-demographic and clinical characteristics.

**A1. Gender** (don’t ask) _____________ **A2. How old were you on your last birthday? ______________**

**A3. What is your occupation? __________________**

**A4. What is the highest level of education you completed? _____________________**

**A5. Are you currently?**  Married Single  Separated Divorced Widowed

**A6. How many persons are there in your household? (not counting you) __________________________**

**A7. Why did you come today in this ambulatory center?** _______________________________

**A8**. **In which of the following activities are you completely independent or you need supervision, direction, personal assistance or total care?**

| **ACTIVITIES** |  | |
| --- | --- | --- |
| Bathing | □ No supervision, direction or personal assistance. | □With supervision, direction, personal assistance or total care |
| Dressing | □ No supervision, direction or personal assistance. | □With supervision, direction, personal assistance or total care |
| Toileting | □ No supervision, direction or personal assistance. | □With supervision, direction, personal assistance or total care |
| Transferring | □ No supervision, direction or personal assistance. | □With supervision, direction, personal assistance or total care |
| Continence | □ No supervision, direction or personal assistance. | □With supervision, direction, personal assistance or total care |
| Feeding | □ No supervision, direction or personal assistance. | □With supervision, direction, personal assistance or total care |

**A9. Which chronic disease(s) do you have?**

| Chronic disease | | Length of the condition |
| --- | --- | --- |
| 1 |  |  |
| 2 |  |  |
| 3 |  |  |
| 4 |  |  |

**Section B.**

This section is designed to gather information about your use of health-care services.

**B1. Which physician usually you consult for a health problem?**

□ general practitioner □ medical specialist □ emergency physician □ hospital physician □ other (specify _______________)

**B2. In the last year, how many times did you visit a general practitioner?** ______ visits

**B3. In the last year, how many times did you visit a medical specialist?** ______ visits (if the answer is 0, skip to B4)

**Which medical specialist? ________________**

| **Medical specialist 1** | **Medical specialist 2** | **Medical specialist 3** |
| --- | --- | --- |
| How many visits? | How many visits? | How many visits? |
|  |  |  |
| For which health concern? | For which health concern? | For which health concern? |
|  |  |  |
|  |  |  |
|  |  |  |

**Which medical specialist? ________________**

| **Medical specialist 1** | **Medical specialist 2** | **Medical specialist 3** |
| --- | --- | --- |
| How many visits? | How many visits? | How many visits? |
|  |  |  |
| For which health concern? | For which health concern? | For which health concern? |
|  |  |  |
|  |  |  |
|  |  |  |

**Which medical specialist? ________________**

| **Medical specialist 1** | **Medical specialist 2** | **Medical specialist 3** |
| --- | --- | --- |
| How many visits? | How many visits? | How many visits? |
|  |  |  |
| For which health concern? | For which health concern? | For which health concern? |
|  |  |  |
|  |  |  |
|  |  |  |

**B4. In the last year, how many times did you go to a hospital emergency department?** ______ times (if the answer is 1 or more, explain the reason(s)) ___________________________________________

**B5. In the last year, how many times were you hospitalized for one night or more?** ______ times (if the answer is 1 or more, explain the reason(s)) ___________________________________________

**Section C.**

This section is designed to gather information about medications for your chronic conditions.

**C1. What medication(s) are you currently taking?**

| Name or description of each medication | How many times a day do you usually take it? | For which condition? | For how long are you taking this medication? |
| --- | --- | --- | --- |
|  |  |  |  |
|  |  |  |  |
|  |  |  |  |
|  |  |  |  |
|  |  |  |  |

**C2. Do your family and friends remind you to take medication on time?**  No (go to C4) Yes, who? _________

**C3. How frequently they remind you to take medication on time?**

always often sometimes rarely

**C4. Do you use a pill box or organiser to help you take your medication?**  No Yes

**C5. Is there any cohabiting who has your chronic condition?**  No Yes, which condition? _______________

**C6. Is there any cohabiting who takes your medication?**  No Yes, which medication? _______________

**Section D.**

This section is designed to gather information about your medications adherence.

**D1. Over the 4 weeks preceding your medical visit:**

1) Do you ever forget to take your medicine? No Yes

2) Are you careless at times about taking your medicine? No Yes

3) Sometimes if you feel worse when you take the medicine, do you stop taking it? No Yes

4) When you feel better do you sometimes stop taking your medicine? No Yes

**Section E.**

This section is designed to assess the sources from which you acquire information about your chronic conditions.

**E1. Do you receive information by the physician regarding the importance of regular clinics checks for your chronic condition(s)?** No Yes

**E2. Do you feel you need more information about the medication(s) for your chronic condition(s)?**  No Yes
